# Rare Manifestation of COVID-19 Resulting in Coronary Artery Vasculitis

**DOI:** 10.1155/2024/8976833

**Published:** 2024-01-30

**Authors:** Ahmed Hassaan Qavi, Soban Ahmad, Neeraj N. Shah, Rony Shammas

**Affiliations:** ^1^Department of Cardiovascular Sciences, East Carolina University Health Medical Center, Greenville, NC, USA; ^2^Department of Cardiovascular Sciences, University of Nebraska Medical Center, Omaha, NE, USA

## Abstract

We present the case of a 59-year-old African American female with end-stage renal disease (ESRD) who presented to the emergency department with chest discomfort. She had a coronary angiogram six months ago that showed no occlusive epicardial coronary artery disease. She had elevated troponin I levels and new regional wall motion abnormalities on echocardiogram. Her SARS-CoV-2 returned positive. After a multidisciplinary team approach, she underwent another coronary angiogram that showed new severe multivessel ostial lesions and a left main coronary artery aneurysm. COVID-19-related coronary artery vasculitis was suspected based on her clinical presentation, angiogram findings, and negative autoimmune workup. The patient underwent successful coronary artery bypass grafting and recovered without complications.

## 1. Introduction

Since December 2019, there has been growing body of research on coronavirus disease 2019 (COVID-19) and incidence of cardiac complications, including ST-elevation myocardial infarction (STEMI), coronary artery dissection, and myocarditis. A postmortem study revealed histologic evidence of left ventricular myocardial necrosis in up to 35% of patients who died of COVID-19 [1]. The proposed pathophysiologic mechanisms of COVID-19-induced acute cardiac syndrome include immune dysregulation, cytokine- and complement-mediated vascular wall injury, and microvascular thrombosis [2]. Coronary arteritis, characterized by coronary stenosis or aneurysm, is typically associated with systemic primary vasculitides like Takayasu Arteritis, polyarteritis nodosa, or Kawasaki disease (KD) [3].

To the best of our knowledge, there are no reported cases of COVID-19-related vasculitis of the coronary arteries. Moreover, only three cases of COVID-19-associated coronary artery aneurysm (CAA) in adults have been reported, and none of them had left main coronary artery (LMCA) involvement [4–6]. Herein, we report a case of left main coronary aneurysm along with new, rapid onset multivessel ostial coronary artery disease (CAD) in an adult end-stage renal disease (ESRD) patient in the setting of COVID-19 infection.

## 2. Case Presentation

**Figure 1:**
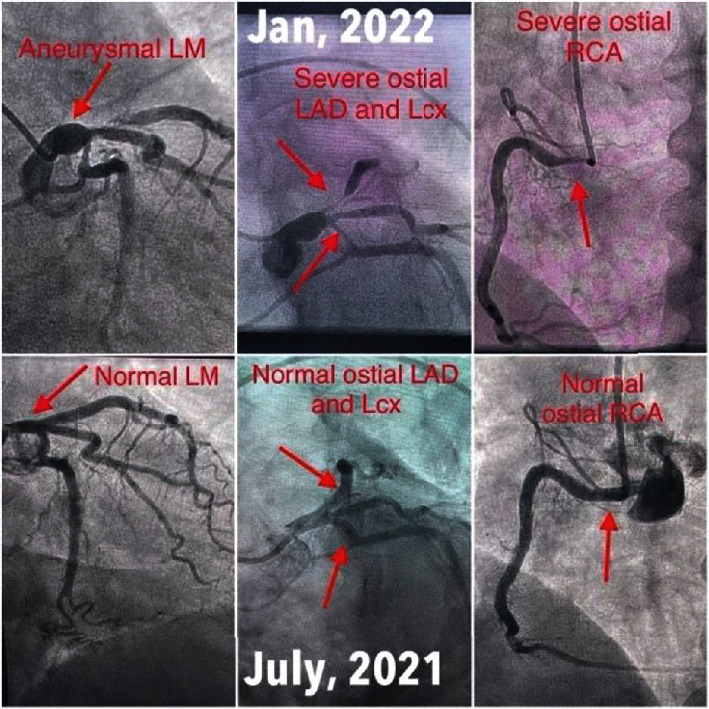
Invasive coronary angiograms showing patent coronary arteries in July 2021 and multivessel ostial coronary artery disease and left main artery aneurysm in January 2022.

A 59-year-old African American female with a past medical history of hypertension, hyperlipidemia, obstructive sleep apnea, and ESRD on hemodialysis presented to the emergency department with pressure-like chest discomfort that initially occurred at rest and resolved spontaneously. The pain recurred on the morning of admission and increased in intensity as she started walking. The progression in her symptoms caused her to seek medical evaluation. She had no known medical history of CAD, congestive heart failure, cardiac or peripheral stents, or stroke. She denied associated shortness of breath, nausea, diaphoresis, palpitations, or syncope. She was a non-smoker and denied illicit substance use. She was being evaluated for kidney transplant, and per preoperative evaluation protocol, she had an invasive coronary angiogram six months ago that showed no occlusive epicardial CAD (Figure 1, July 2021, and Video 1).

On physical examination, she was afebrile, blood pressure was 113/83 mmHg, heart rate was 67 beats per minute, and respiratory rate was 16 breaths per minute. Her body mass index was 39.4 kg/m^2^. Physical examination was unremarkable.

Her initial electrocardiogram (ECG) showed a normal sinus rhythm with no ischemic ST-T wave changes, unchanged compared to prior ECG six months ago. Her complete blood count showed mild leukocytosis (11.4 × 10^9^/L) and no anemia or thrombocytopenia. Her blood chemistry showed a sodium of 135 mmol/L, potassium 4.2 mmol/L, bicarbonate 31 mmol/L, BUN 56 mg/dL, creatinine 8.7 *μ*mol/L, calcium 10.2 mmol/L, phosphorus 4.2 mmol/L, and magnesium 2.0 mmol/L. Her first troponin I was 0.15 ng/mL (normal < 0.03 ng/mL). Second and third troponins I checked at 2- and 8-hour intervals were 0.52 ng/mL and 0.63 ng/mL, respectively. Her chest X-ray was negative for any pulmonary infiltrates.

At admission, she tested positive for SARS-CoV-2. She did recall exposure to a COVID-19 patient during a hemodialysis session one week ago. Of note, she received COVID-19 immunization booster two months ago. She did not exhibit any classic respiratory or systemic symptoms associated with COVID-19.

She also underwent a thromboembolic workup. Her D-dimer returned negative, and she underwent computed tomography angiogram of her chest and ultrasound Doppler of both lower extremities, both tests returning negative for pulmonary embolism and deep vein thrombosis, respectively.

Transthoracic echocardiogram showed left ventricular ejection fraction (LVEF) 50-55% with new regional wall motion abnormalities in the anterior, apical, and inferior walls suggestive of multivessel territory ischemia. She was diagnosed and appropriately treated for non-ST-elevation myocardial infarction and underwent repeat coronary angiogram under strict COVID-19 isolation precautions after multidisciplinary team discussion.

## 3. Workup

**Figure 2:**
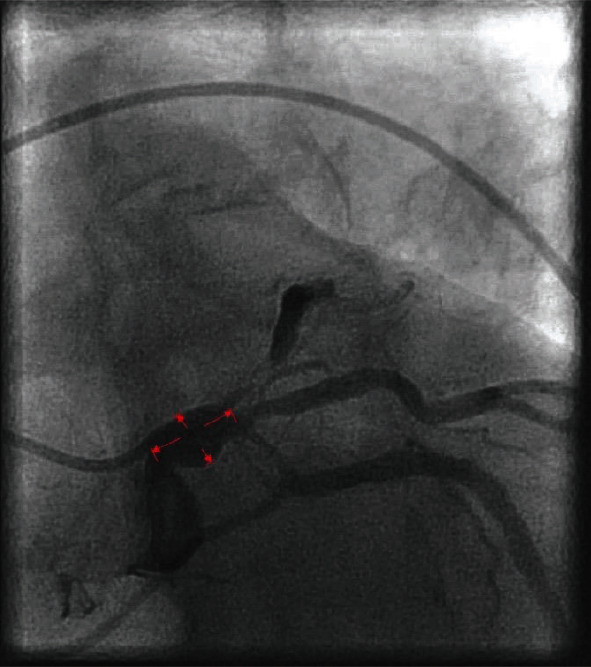
Left main artery aneurysm measuring 2.5 × 4.0 cm.

Invasive coronary angiography showed new-onset severe multivessel epicardial CAD (Figure 1, January 2022, and Video 2). Her LMCA was aneurysmal (2.5 × 4.0 cm) (Figure 2), ostial left anterior descending artery (LAD) had 95% tubular stenosis with thrombolysis in myocardial infarction- (TIMI-) 2 flow distally, and left circumflex artery (LCX) had tubular 80-90% ostial to proximal stenosis with TIMI-2 flow distally. Right coronary artery (RCA) was the dominant vessel with tubular 80-90% ostial stenosis. These findings were new compared to six months ago. Based on her clinical presentation and coronary angiogram, coronary artery vasculitis was suspected. An exhaustive autoimmune workup was performed. Erythrocyte sedimentation rate and C-reactive protein were elevated at 130 mm/h and 57.8 mg/L, respectively. Antinuclear antibody was negative, and complement C3 and C4 levels were normal (148 mg/dL and 46 mg/dL, respectively). Antineutrophilic cytoplasmic (c-ANCA and p-ANCA) and double-stranded DNA antibodies were negative. Glomerular membrane antibody was also negative. Human immunodeficiency viruses 1 and 2, Cytomegalovirus, Toxoplasma gondii, syphilis, and Epstein-Barr virus antibodies were all negative.

## 4. Treatment

Given ongoing chest pain and borderline hypotension, patient underwent intra-aortic balloon pump placement in the cardiac catheterization laboratory and was transferred to cardiac intensive care unit. She was evaluated by cardiothoracic surgery and underwent coronary artery bypass grafting (CABG) two days later. She received a left internal mammary artery (LIMA) graft to her LAD and a saphenous vein graft (SVG) to the second obtuse marginal (OM2) branch. The posterior descending artery was an acceptable target but was not bypassed due to a paucity of conduit. Radial arteries were not a viable option due to renal failure and fistulas. Right IMA was not used due to patient's significant risk for sternal wound complications. Her bilateral below the knee lower extremity veins had old, healed wounds preventing their use. Saphenous vein was aneurysmal in several segments and 8 mm in size creating a significant size discrepancy between her coronary arteries and the vein conduit. Only one portion of the saphenous vein was viable and sufficient to bypass the left circumflex system. The distal segment of the IMA was sent for a histopathologic examination due to suspicion of vasculitis. Histopathology of LIMA showed normal arterial architecture without any pathologic changes suggestive of vasculitis. Coronary artery samples (LAD or LCX) were not sent for pathology by the operating surgeon, recognizing the challenges of preserving small coronary vessel segments during surgery and concern for obtaining adequate tissue for an accurate histopathologic examination. She fared well postoperatively and was discharged to a rehabilitation facility.

**Figure 3:**
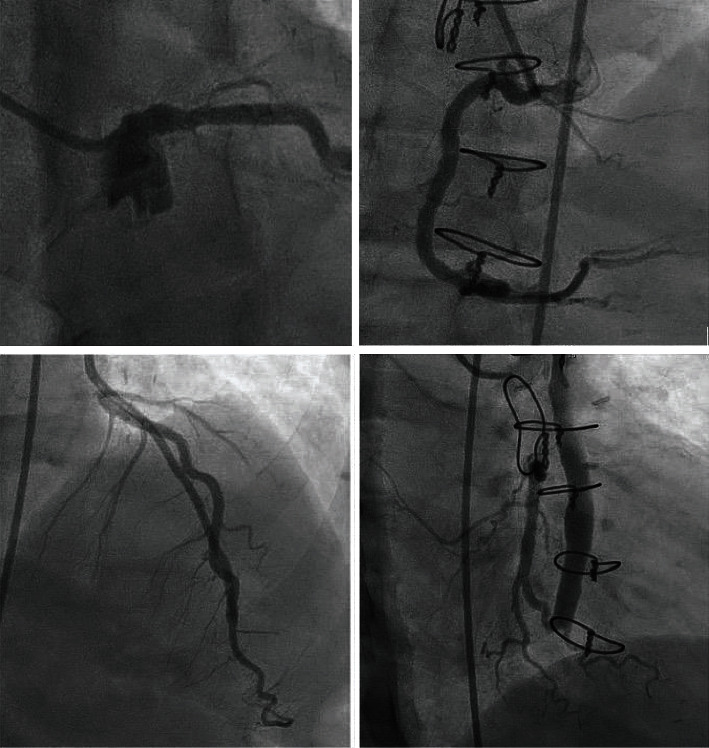
Follow-up coronary and bypass angiogram showing persistent left main artery aneurysm and patent left internal mammary artery to left anterior descending artery graft and patent saphenous vein to obtuse marginal artery graft.

She remained asymptomatic at one-year follow-up; however, nuclear stress test (done for prerenal transplant evaluation) showed a new medium-sized anterior myocardial wall defect with normal poststress ejection fraction of 60%. Considering the patient's lack of symptoms and preserved ejection fraction, the clinical concern for myocarditis or cardiomyopathy was low. Nevertheless, given her history and upcoming renal transplant, repeat coronary angiography was pursued (14 months after index hospitalization) for a comprehensive assessment. It revealed a persistent left main CAA and widely patent LIMA to LAD and SVG to OM2 grafts (Figure 3 and Video 3). The native LAD and LCX were both 100% occluded, and RCA had unchanged ostial disease.

## 5. Discussion

The presence of multiple coronary ostial lesions and the CAA suggests a vasculitic process [7]. The histopathological evaluation of coronary artery vasculitis can be challenging due to logistical difficulties in obtaining a coronary artery biopsy [8]. Biopsy of adjacent vasculature, such as the IMA, may appear normal but can still hold prognostic value when harvested for CABG, as demonstrated in our patient's case. Thus, diagnosis of coronary artery vasculitis is primarily based on a combination of clinical presentation, elevated inflammatory markers, multimodality imaging, and coronary angiography findings [8]. Recently, KD-like coronary vasculitis has been reported in adult and pediatric patients with COVID-19 [4]. Interestingly, KD-like vasculitis has been observed in patients without active COVID-19 pneumonia [9]. Similarly, our patient did not have any acute viral illness-like symptoms or evidence of COVID-19 pneumonia which makes her presentation likely related to an underlying immunologic process related to COVID-19 that is supported by markedly elevated inflammatory markers.

The exact mechanism of CAAs and ostial lesions in COVID-19 is unknown, but possible mechanisms include either virus-triggered immune response or direct viral damage to the coronary vasculature leading to accelerated atherosclerosis, localized collagenase activation in atheroma, endothelial dysfunction, and hypercoagulability [10]. Our patient did not have any manifestation of primary vasculitis. Her ESRD resulted from uncontrolled hypertension, and she did not have chronic symptoms that might point to vasculitis, including skin rash, chronic anemia, thrombocytopenia, or chronically elevated inflammatory markers. Patients with ESRD are at higher risk for atherosclerosis, the most common cause of CAA. However, coincidentally, our patient had a normal coronary angiogram six months ago (as a part of prerenal transplant workup). This, in addition to positive COVID-19 test and symptom onset one week after COVID-19 exposure, suggested that COVID-19-related coronary vasculitis was the likely diagnosis. Patients with ESRD may be at increased risk of coronary events from COVID-19 and thus have a worse prognosis.

CAA is characterized as 1.5-fold localized dilation of the coronary artery compared to the nearby normal coronary segments. Common causes of CAA include advanced atherosclerosis, KD and other rheumatologic processes, mycotic infections, and congenital lesions. The management of CAA is usually based on the concomitant presence of obstructive coronary disease, the location and size of the aneurysm, aneurysmal expansion, and associated infectious etiologies. Treatment options include surgical, percutaneous, or medical interventions. Our literature review yielded only three other documented case reports of COVID-19-related CAA.  Table 1 summarizes the data on patient characteristics, clinical features, diagnostic studies, and management of CAA in the context of COVID-19.

## 6. Conclusion

We report a rare case of accelerated multivessel CAD and new left main CAA in the setting of COVID-19 infection due to presumed COVID-19-related vasculitis that was confined to the epicardial coronary bed for unclear reasons. Patients with ESRD may be at increased risk for accelerated atherosclerosis and immune-related coronary vascular manifestations.

## Figures and Tables

**Table 1:** Comparison with the prior reported cases of coronary artery aneurysm related to COVID-19.

Authors	Diakite et al. [4]	Alao et al. [5]	Shin et al. [6]	Qavi et al.
Publication year	2021	2022	2022	Present case
Race	Sub-Saharan African	Pakistani	Not reported (case reported from Korea)	African American
Age (years)/gender	33/M	35/M	66/M	59/F
Immunologic workup	Negative	None	None	Negative
ESRD	No	No	Yes	Yes
Clinical presentation	Fever, dyspnea, chest pain, shock	Central chest pain radiating to left arm	Chest pain	Chest pain
ECG	No ST segment abnormalities	Inferior ST segment elevation	Not reported	No ST segment abnormalities
Troponin	Initial troponin 13.2 ng/mL. No trend was reported	Initial troponin T 0.009 ng/mL. No trend reported	Not reported	Initial troponin I was 0.15 ng/mL, followed by 0.52 ng/mL and 0.63 ng/mL at two and eight hours, respectively
C-reactive protein	150 mg/L	Not reported	Normal	57.8 mg/L
Echo	LVEF 20% with global hypokinesia	Not reported	LVEF 40% with hypokinesia of the base to mid inferior wall and basal inferolateral wall and akinesia of the mid inferolateral wall	LVEF 50-55%, new RWMA in the anterior, apical, and inferior walls
COVID-19 vaccination status	Not reported	Not reported	Received first dose 4 weeks prior	Received booster two months prior
COVID19 symptoms/testing	Viral symptoms started six weeks prior to presentation. Testing showed +ve IgG but –ve PCR	Asymptomatic/testing was +ve during the same admission	Hospitalization with COVID-19 pneumonia 4 months prior	Asymptomatic/testing showed both +ve PCR and +ve IgG
COVID19 pneumonia	Not reported	Chest X-ray was normal	Not reported	Chest X-ray was normal
Coronary angiogram	Coronary CT angiogram: multiple aneurysms involving the RCA, intraventricular artery, and LCX	Coronary angiogram: large proximal RCA aneurysm (14.46 mm diameter) with occlusive thrombus. Medium-sized LCX aneurysm	Coronary angiogram: proximal RCA aneurysm with a diameter of 8.6 mm. Multiple stenotic lesions in the RCA and LCX	Coronary angiogram: aneurysmal left main artery (4 × 9 mm), ostial LAD was subtotally occluded, LCX 90% eccentric ostial stenosis. Dominant RCA with 70-80% ostial stenosis
Treatment	Medical management with IVIG, prednisone, and aspirin	Clot removed with an aspiration catheter	2v-CABG: SVG to RCA, SVG to OM1 along with aneurysmal suturing	2v-CABG: LIMA to LAD, SVG to OM2
Patient outcome	At 5-month follow-up, repeat coronary CT showed complete resolution of CAA	At 2-month follow-up, the patient had no cardiac complications	At 5-month follow-up, the patient has no cardiac complications	At one-year follow-up, patient was asymptomatic. However, nuclear stress test showed a new anterior myocardial wall defect

Abbreviations: LVEF: left ventricular ejection fraction; IgG: immunoglobulin G; PCR: polymerase chain reaction; RWMA: regional wall motion abnormality; CT: computer tomography; RCA: right coronary artery; LCX: left circumflex artery; LAD: left anterior descending artery; OM: obtuse marginal; CABG: coronary artery bypass grafting; SVG: saphenous vein graft; CAA: coronary artery aneurysm; IVIG: intravenous immunoglobulin.

## Supplementary Materials

Supplementary Materials Video 1: first invasive coronary angiogram six months prior to presentation showing no occlusive epicardial coronary artery disease. Video 2: second invasive coronary angiography showing new-onset severe multivessel epicardial coronary artery disease. Video 3: repeat invasive coronary angiography (14 months after the index hospitalization) demonstrating a persistent left main coronary artery aneurysm and widely patent LIMA to LAD and SVG to OM2 grafts.

## References

[1] Pellegrini D., Kawakami R., Guagliumi G. (2021). Microthrombi as a major cause of cardiac injury in COVID-19: a pathologic study. *Circulation*.

[2] Madjid M., Safavi-Naeini P., Solomon S. D., Vardeny O. (2020). Potential effects of coronaviruses on the cardiovascular system: a review. *JAMA Cardiology*.

[3] Gori T. (2021). Coronary Vasculitis. *Biomedicines*.

[4] Diakite S., Bousdira N., Tachon G., Ackermann F., Groh M., Rohmer J. (2021). Regression of coronary aneurysms with intravenous immunoglobulins and steroids for COVID-19 adult multisystem inflammatory syndrome. *JACC: Case Reports*.

[5] Alao D. O., Alabdouli A., Jalabi A. (2022). Coronary artery aneurysm presenting as ST-elevation myocardial infarction in a man with coronavirus disease 2019: a case report. *Journal of Medical Case Reports*.

[6] Shin J. H., Ro S. K. (2022). Newly diagnosed right coronary artery aneurysm in an adult with recent coronavirus disease 2019 infection. *Diagnostics*.

[7] Abou Sherif S., Ozden Tok O., Taşköylü Ö., Goktekin O., Kilic I. D. (2017). Coronary artery aneurysms: a review of the epidemiology, pathophysiology, diagnosis, and treatment. *Frontiers in Cardiovascular Medicine*.

[8] Pagnoux C., Guillevin L. (2005). Cardiac involvement in small and medium-sized vessel vasculitides. *Lupus*.

[9] Jiang L., Tang K., Levin M. (2020). COVID-19 and multisystem inflammatory syndrome in children and adolescents. *The Lancet Infectious Diseases*.

[10] Esposito L., Cancro F. P., Silverio A. (2021). COVID-19 and acute coronary syndromes: from pathophysiology to clinical perspectives. *Oxidative Medicine and Cellular Longevity*.

