# Supplementary material for: Rare Manifestation of COVID-19 Resulting in Coronary Artery Vasculitis
Source: Case Rep Cardiol. 2024 Jan 30;2024:8976833. doi: 10.1155/2024/8976833 (PMC10846917; doi:10.1155/2024/8976833)
Supplement: Supplementary Materials — Video 1: first invasive coronary angiogram six months prior to presentation showing no occlusive epicardial coronary artery disease. Video 2: second invasive coronary angiography showing new-onset severe multivessel epicardial coronary artery disease. Video 3: repeat invasive coronary angiography (14 months after the index hospitalization) demonstrating a persistent left main coronary artery aneurysm and widely patent LIMA to LAD and SVG to OM2 grafts. [file 8976833.f1.docx]

**Supplementary material**

**Video 1:** [**https://drive.google.com/file/d/1wk1iujognOBG9w2f8semkfLO1LpVKIty/view?usp=share_link**](https://drive.google.com/file/d/1wk1iujognOBG9w2f8semkfLO1LpVKIty/view?usp=share_link)

Caption: First invasive coronary angiogram six months prior to presentation showing no occlusive epicardial coronary artery disease

**Video 2:**

[**https://drive.google.com/file/d/1nHHlCFGgk1L7mSMMmffCjwCzSQZe8cDq/view?usp=share_link**](https://drive.google.com/file/d/1nHHlCFGgk1L7mSMMmffCjwCzSQZe8cDq/view?usp=share_link)

Caption: Second invasive coronary angiography showing new onset severe multivessel epicardial coronary artery disease.

**Video 3:**

[**https://drive.google.com/file/d/1_lL2L0ocOmod6Odt8bpKv_xIMVFRkb5i/view?usp=share_link**](https://drive.google.com/file/d/1_lL2L0ocOmod6Odt8bpKv_xIMVFRkb5i/view?usp=share_link)

Caption: Repeat invasive coronary angiography (14 months after the index hospitalization) demonstrating a persistent left main coronary artery aneurysm and widely patent LIMA to LAD and SVG to OM2 grafts.
